# Methane oxidation and methylotroph population dynamics in groundwater mesocosms

**DOI:** 10.1111/1462-2920.14929

**Published:** 2020-02-07

**Authors:** Olukayode Kuloyo, S. Emil Ruff, Aaron Cahill, Liam Connors, Jackie K. Zorz, Isabella Hrabe de Angelis, Michael Nightingale, Bernhard Mayer, Marc Strous

**Affiliations:** ^1^ Department of Geoscience University of Calgary Calgary Alberta Canada; ^2^ Shell International Exploration and Production Inc Westhollow Technology Center Houston TX USA; ^3^ Ecosystems Center Marine Biological Laboratory Woods Hole MA USA; ^4^ The Lyell Centre Heriot Watt University Edinburgh United Kingdom; ^5^ Biomedical Sciences Department, Faculty of Medicine University of Calgary Calgary Alberta Canada; ^6^ Multiphase Chemistry Department, Max Planck Institute for Chemistry Mainz Germany

## Abstract

Extraction of natural gas from unconventional hydrocarbon reservoirs by hydraulic fracturing raises concerns about methane migration into groundwater. Microbial methane oxidation can be a significant methane sink. Here, we inoculated replicated, sand‐packed, continuous mesocosms with groundwater from a field methane release experiment. The mesocosms experienced thirty‐five weeks of dynamic methane, oxygen and nitrate concentrations. We determined concentrations and stable isotope signatures of methane, carbon dioxide and nitrate and monitored microbial community composition of suspended and attached biomass. Methane oxidation was strictly dependent on oxygen availability and led to enrichment of ^13^C in residual methane. Nitrate did not enhance methane oxidation under oxygen limitation. Methylotrophs persisted for weeks in the absence of methane, making them a powerful marker for active as well as past methane leaks. Thirty‐nine distinct populations of methylotrophic bacteria were observed. Methylotrophs mainly occurred attached to sediment particles. Abundances of methanotrophs and other methylotrophs were roughly similar across all samples, pointing at transfer of metabolites from the former to the latter. Two populations of Gracilibacteria (Candidate Phyla Radiation) displayed successive blooms, potentially triggered by a period of methane famine. This study will guide interpretation of future field studies and provides increased understanding of methylotroph ecophysiology.

## Introduction

Oil and natural gas extraction from organic‐rich shale formations has transformed the global energy outlook (Malakoff, 2014). More than 100,000 of oil and gas wells completed in the United States and Canada over the past decade were horizontally drilled and hydraulically fractured (Kerr, 2010; McIntosh *et al*., 2019). In some of these wells, well bore integrity failure leads to the unintentional subsurface release of natural gas—also known as fugitive methane or stray gas (Vidic *et al*., 2013; Darrah *et al*., 2014). Such release may be followed by gas migration via multiphase fluid flow, through geological profiles, toward groundwater and the water‐unsaturated vadose zone, ultimately resulting in atmospheric emissions (Cahill *et al*., 2019). Methane, the main component of natural gas, has a global warming potential 86 times greater than CO_2_ over 20 years, and 25 times greater over 100 years (Shindell *et al*., 2009; Frankenberg *et al*., 2011).

During migration, methane may be oxidized by methanotrophic and methylotrophic Bacteria and Archaea inhabiting the groundwater. Methylotrophs are microorganisms oxidizing compounds with a methyl (‐CH_3_) group, such as methane and methanol. Methanotrophs refers to the subgroup of methylotrophs capable of methane oxidation. In freshwater and marine environments microbial methane oxidation is known to be a critical methane sink that limits methane emissions (Le Mer and Roger, 2001; Knittel *et al*., 2005). Methane oxidation may proceed aerobically in the presence of oxygen, or anaerobically with nitrate, sulphate, and oxidized forms of iron and manganese (Conrad, 1996; Hanson and Hanson, 1996; Boetius *et al*., 2000; Orphan *et al*., 2002; Ettwig *et al*., 2010; Haroon *et al*., 2013; Ettwig *et al*., 2016; Cai *et al*., 2018). Aerobic oxidation of methane may lead to increased turbidity resulting from microbial growth, oxygen limitation, anoxic conditions (Cahill *et al*., 2017), and, in theory, production of sulfide by microbial sulfate reduction. Thus, while bioremediation may limit methane emissions to the atmosphere, it may also reduce groundwater quality (Révész *et al*., 2010; Osborn *et al*., 2011). Under oxygen‐limiting conditions, aerobic methanotrophs may shift to partially anaerobic respiration using nitrate (Hoefman *et al*., 2014; Kits *et al*., 2015; Heylen *et al*., 2016). Methanotrophs may also leak out metabolites, such as methanol or acetate, which are then further oxidized by other methylotrophic bacteria, which may use nitrate as electron acceptor (Nercessian *et al*., 2005; Chistoserdova *et al*., 2009; Takeuchi, 2019). In a recent controlled natural gas injection field experiment, microbial methane oxidation was shown to be strictly dependent on oxygen (Cahill *et al*., 2017, 2018; Steelman *et al*., 2017; Forde *et al*., 2019). No anaerobic oxidation of methane was observed, and a lack of oxygen led to persistent (i.e. up to 700 days post injection) presence of methane in the aquifer.

Even though methane is often detected in groundwater with reducing redox conditions (Darling and Gooddy, 2006; Gorody, 2012; Humez *et al*., 2016), the literature on methane oxidation in groundwater is limited compared to marine and freshwater sediments and mostly reliant on geochemical and isotopic analyses of groundwater gas and water samples (Van Stempvoort *et al*., 2005; Cheung *et al*., 2010; Jackson *et al*., 2013; Humez *et al*., 2015; Humez *et al*., 2016). Methane of biogenic origin, which may have migrated into groundwater, or was produced *in situ* by Archaea naturally present in the aquifer, can be distinguished from thermogenic methane, by its isotopic composition since biogenic methane is more isotopically depleted in ^13^C (δ^13^C typically between ‐50‰ and ‐110‰, relative to Vienna Pee Dee Belemnite, VPDB) than thermogenic methane (δ^13^C typically between ‐25‰ and ‐55‰) (Whiticar, 1999). However, interpretations of isotope compositions are not always straightforward, because if methane is being oxidized by microbes, remaining methane may become more enriched in δ^13^C, leading to a pseudo‐thermogenic signature (Whiticar, 1999). This ^13^C enrichment in the remaining methane is caused by a slight preference of methanotrophs for the lighter (^12^C) isotope.

Field studies have a number of other limitations. Most or all samples for microbial analysis come in the form of water from wells, whereas a large part of the groundwater bacteria may be attached to particles or sediments within the subsurface and therefore remain invisible. Furthermore, groundwater flow and gas migration in the subsurface are not homogeneous and therefore, environmental conditions are partially unknown, adding uncertainty to statistical inferences about relationships between environmental conditions and the occurrence of bacteria of interest. Groundwater microbial communities harbor members of the Candidate Phyla Radiation and other unknown bacteria that may affect the fitness of methylotrophs by antagonistic ecological interactions (Brown *et al*., 2015; Anantharaman *et al*., 2016; Cross *et al*., 2019). In the present study, we address these issues using laboratory mesocosms inoculated with groundwater from our previous field injection experiment (Cahill *et al*., 2017).

Five sets of triplicated mesocosms were run for 35 weeks to investigate how different environmental aquifer conditions selected for specific methanotrophic and methylotrophic populations and affected methane bioremediation outcomes. We also investigated the potential for nitrate to serve as an alternate electron acceptor for methane oxidation, persistence of methanotrophs during famine periods and the effect of methane bioremediation on stable isotope fingerprints of methane, nitrate and carbon dioxide. We used 16S rRNA gene amplicon sequencing and cell counting to quantify the abundance of methanotrophic and associated bacteria in response to dynamic methane and oxygen regimes, of both attached and suspended biomass. Our study will assist interpretation of future field studies on microbial methane bioremediation in groundwater and provides new insights on the ecophysiology of methylotrophic bacteria with regard to oxygen, methane and nitrate concentrations.

## Results

### *Mesocosms and inoculation*

**Figure 1:**
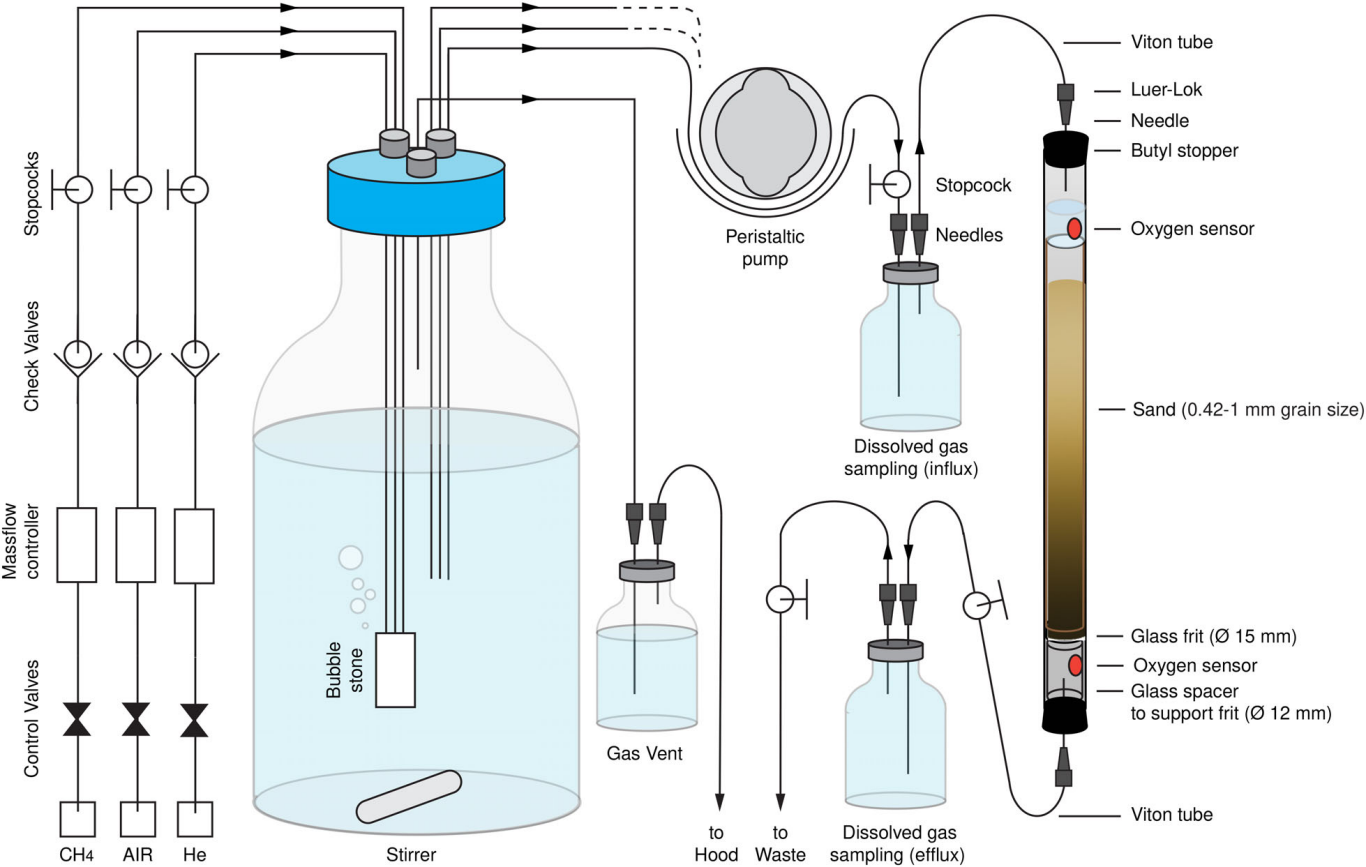
Schematic representation of the mesocosm experimental setup. [Color figure can be viewed at http://wileyonlinelibrary.com]

To explore methane bioremediation potential and microbial community response to different stray gas leakage scenarios, five sets of triplicated, continuous flow, laboratory mesocosm experiments were set up (Fig. 1). All mesocosms, static sand columns perfused with a continuous flow of medium, were inoculated with groundwater obtained from a previous field methane release experiment (Cahill *et al*., 2017). Seven groundwater samples obtained from two wells located immediately downstream of the methane release site, sampled at depths between 2 and 8 m and between 55 and 333 days after the start of methane release, were mixed and used to inoculate all fifteen mesocosms simultaneously to ensure a homogeneous distribution of microbial diversity.

The microbial community in the groundwater, in the water flowing out of the mesocosms and attached to mesocosm sediment particles, was profiled with 16S rRNA gene amplicon sequencing. Across all 234 samples, 4,486 unique amplicon sequence variants (ASVs) were recovered (Supplementary data S1). Thirty‐nine ASVs were affiliated with ten known methylotrophic genera and were observed in at least three samples. Representatives of ten different methylotrophic genera, affiliated with Alpha‐, Beta‐ and Gammaproteobacteria, were detected (Table 1), including five methanotrophic genera. Because the amplicon sequences were quite short (~400 nucleotides), classification beyond the level of genus was generally not feasible. Forty‐three ASVs were affiliated with the Candidate Phyla Radiation. Two of those, both affiliated with Gracilibacteria (BD1‐5/SNO2), were occasionally quite abundant (Table 1). Table 1 also lists two genera, represented by 51 ASVs, that were used as marker taxa for anoxic conditions: *Pelosinus* and *Desulfosporosinus* (Beller *et al*., 2013; Shelobolina *et al*., 2007). Known anaerobic methane oxidizers were only detected sporadically, in very few samples and at extremely low abundance. Therefore, it appears that methane oxidation in the mesocosms was strictly aerobic, as was observed previously in the field (Cahill *et al*., 2017).

**Table 1:** Taxonomic affiliation and statistics of key amplicon sequence variants (ASVs). Supplementary Data S1 lists taxonomic classifications, sequences and abundances of all ASVs across all samples.

Physiology	Genus	Class	# ASVs obser‐ved	Field average abun‐dance (%)	Mesocosm average abundance (%)	Mesocosm maximum abundance (%)	#observations	ASVs
Methanotroph	Methylocystis/sinus^a^	Alpha‐proteobacteria	3	0.0	2.4	32.3	196	15, 23, 82
Methanotroph	Methylobacter	Gamma‐proteobaceria	5	14.5	0.1	6.1	56	55, 124, 272, 444, 1051
Methanotroph	Methylovulum	Gamma‐proteobaceria	6	0.4	0.4	19.8	153	34, 40, 167, 402, 592, 1057
Methanotroph	Methylomonas	Gamma‐proteobaceria	2	0.0	0.1	24.0	19	89, 187
Methanotroph	Crenothrix	Gamma‐proteobaceria	2	0.0	0.0	0.8	11	437, 823
Methylotroph	Hyphomicro‐bium	Alpha‐proteobacteria	8	0.0	0.2	4.6	113	88, 228, 424, 853, 1115, 1227, 1831, 3223
Methylotroph	Methylo‐bacterium	Alpha‐proteobacteria	3	0.0	0.0	1.0	24	533, 710, 875
Methylotroph	Methylo‐versatilis	Beta‐proteobacteria	1	0.0	2.1	29.4	194	14
Methylotroph	Methylotenera	Beta‐proteobacteria	6	2.1	0.2	3.6	122	106, 190, 327, 539, 646, 945
Methylotroph	Methylophilus	Beta‐proteobacteria	3	3.0	0.1	13.5	14	111, 153, 1552
Unknown	Gracilibacteria	Candidate Phyla Radiation	2	0.0	0.6	12.1	150	24, 74
Fermentation	Pelosinus	Negativicutes	11	0.0	2.3	86.4	94	10, 19, 707, 1452, 1592, 2442, 2550, …
Sulfate reduction	Desulfosporosinus	Clostridia	40	2.4	0.3	19.8	162	46, 351, 374, 381, 382, 457, 525, 724, …

a These two genera could not be discriminated based on the 400 nucleotide amplicon.

Amplicon sequencing showed that after inoculation, the microbial communities present in each mesocosm were similar to each other, but different from the groundwater in the field. For example, whereas bacteria related to the Gammaproteobacterium *Methylobacter* were the most abundant methanotrophs *in situ* (up to 41 % relative sequence abundance), bacteria affiliated with the Alphaproteobacteria *Methylocystis* or *Methylosinus* were most abundant in the mesocosms directly after inoculation (up to 11%).

This shift might be explained by differences in resilience across populations during 9‐14 months of storage of the groundwater between sampling and inoculation or by differences in the ability to attach to sand particles during the inoculation itself. *Methylobacter* did persist in the mesocosms and was the most abundant methanotroph in some samples. Methylotrophic Alphaproteobacteria such as *Methylocystis* are considered more resilient during harsh conditions than methylotrophic Gammaproteobacteria such as *Methylobacter* (Ho *et al*., 2012; Knief, 2015).

The first set of triplicated mesocosms was perfused with medium containing dissolved methane (up to 0.4 mM) and oxygen (up to 0.15 mM). In the second set of mesocosms, the medium contained nitrate (up to 0.3 mM), in addition to dissolved methane and oxygen. The third set was perfused with anoxic medium with dissolved methane and nitrate. The fourth set received anoxic media with dissolved methane only. The final set received medium without any of these additions.

Profiling the microbial community of cells suspended in the medium flowing out of the mesocosms was performed at twelve time points during the 35 week experiments. Profiling the microbial community attached to sediments was much more disruptive, because mesocosms needed to be opened and sediment removed. This was only done three times throughout the experiment.

## Mesocosm biogeochemistry

**Figure 2:**
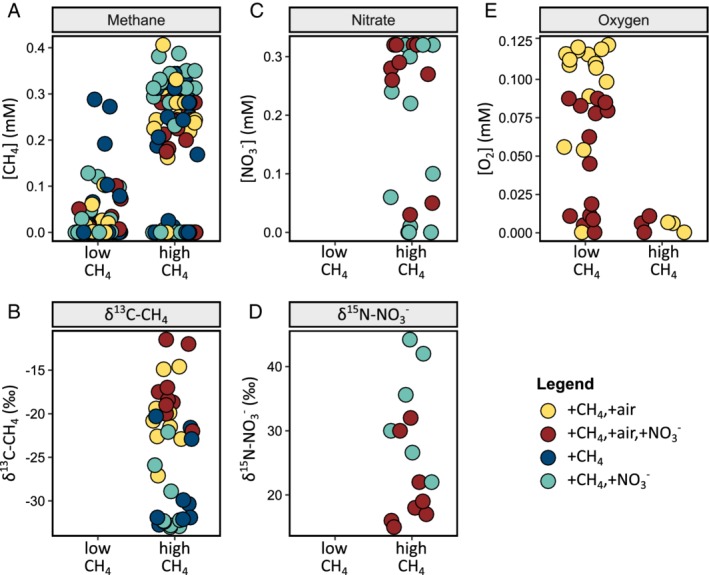
Environmental conditions during the periods of low and high methane supply in four sets of triplicated mesocosms (the fifth set did not receive any methane, nitrate or air). The legend shows conditions and colors. A, The methane concentration in medium flowing out of the mesocosms. B, δ13C of the residual methane. C, Nitrate concentration in outflowing medium. D, δ15N of residual nitrate. E, Oxygen concentration in outflowing medium. Nitrate and isotope composition were only measured during the high methane phase. Each dot represents one sample, with values tabulated in Supplementary Data S1. [Color figure can be viewed at http://wileyonlinelibrary.com]

During the first ten weeks, the methane concentration in the medium flowing into the mesocosms was 0.4 mM. The medium flow rate was 100 ml day^‐1^, equivalent to a water velocity of 1.8 m day^‐1^. During this “low CH_4_” period, those mesocosms supplied with dissolved air generally displayed complete methane consumption (Fig. 2A). They also displayed residual oxygen in the outflowing medium (Fig. 2E), indicating that those mesocosms were generally oxic. In mesocosms without dissolved air, the methane concentrations in the outflowing medium were higher, but still lower than in the inflowing medium, indicating some methane consumption in these experiments. Given the absence of known anaerobic methanotrophs in our amplicon data set, methane consumption in these mesocosms can most easily be explained by assuming some oxygen ingress, for example via rubber tubing (Fig. 1), into these mesocosms. Nitrate was not measured during the “low CH_4_” period (Fig. 2C).

After ten weeks, methane was removed from the medium of all mesocosms, for seven weeks, enabling us to assess persistence of methylotrophic bacteria in the absence of methane.

Seventeen weeks after the start of the experiment, methane (~1 mM) was reintroduced into the medium, and the medium flow rate was gradually increased to 200 ml day^‐1^, equivalent to a water velocity of 3.6 m day^‐1^. During this 16‐week “high CH_4_” period, methane was present in excess (Fig. 2A), and oxygen limitation occurred (Fig. 2E). Methane was detected in the outflowing medium at a concentration up to 0.4 mM. In the presence of oxygen, the δ^13^C value of this residual methane was between ‐27 ‰ and ‐12 ‰ (Fig. 2B), more than 10 ‰ higher than the δ^13^C value of methane of ‐36 ‰ in the inflowing medium. At the same time, the δ^13^C value of the dissolved carbon dioxide decreased from ‐28‰ in the inflowing medium to below ‐43 ‰ in the outflowing medium (Supplementary Data S1). ^13^C enrichment in residual methane and ^12^C enrichment in dissolved carbon dioxide were much lower in mesocosms without oxygen, indicating that less methane was oxidized in those experiments.

Mesocosms provided with nitrate displayed nitrate consumption, reproduced across replicates, especially in experiments that did not receive dissolved air (Fig. 2C). Consistently, the δ^15^N value of nitrate increased from 15 ‰ in the inflowing medium, up to 44 ‰ in the outflowing medium (Fig. 2D). The rate of nitrate consumption varied, and it was not observed at all time points. Concentrations and isotopic compositions of methane, carbon dioxide and nitrate were internally consistent across all samples. For example, samples with lower methane concentrations showed higher enrichment of ^13^C in residual methane, as well as higher carbon dioxide concentrations and higher enrichment of ^12^C in produced carbon dioxide. Samples with lower nitrate concentrations showed higher enrichment of ^15^N in the residual nitrate.

### *Methylotrophs attached to sediment particles*

**Figure 3:**
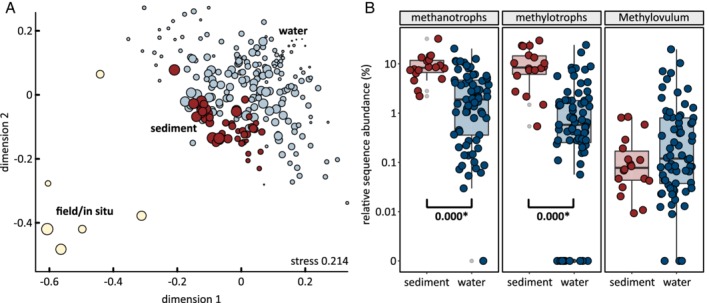
| Comparison of sediment and water communities. A, Nonmetric multidimensional scaling (NMDS) plot showing the sediment community (red) was more stable across experiments and time than the water community (light blue). Size of bubbles shows Shannon entropy. Richness of the sediment communities (135±68 ASVs, Shannon 3.1±0.8) was higher than of the water communities (102±31 ASVs, Shannon 2.6±0.7), but lower than in the field (175±82 ASVs, Shannon 3.6±0.9). B, Relative sequence abundance of methanotrophs (18 ASVs detected) and all other methylotrophs (21 ASVs detected) was higher in sediment communities than water communities (Wilcoxon rank sum test). P values of significant differences between water and sediment abundances are shown. All methylotrophic bacteria showed higher sequence abundance in sediment communities, except for ASVs affiliated with *Methylovulum*. [Color figure can be viewed at http://wileyonlinelibrary.com]

The outflowing medium of all mesocosms contained 1.3±0.3·10^5^ cells ml^‐1^ (SD, *n* = 30) in the “low CH_4_” phase of all experiments (Supplementary Data S1). Cell counts of suspended cells peaked at 5.1·10^5^ and 3.8·10^5^ cells ml^‐1^ during the “high CH_4_” phase of mesocosms with and without dissolved air respectively. An additional unknown number of cells inhabited the mesocosms attached to sediment particles. Attached cells were sampled for community profiling during week 10, at the end of the “low CH_4_” phase and in weeks 31 and 35, at the end of the “high CH_4_” phase and during the last methane‐famine phase. The sediment communities displayed more diversity than the suspended communities, were more stable in time and more similar across replicates and treatments (Fig. 3). They also displayed two to twenty times higher relative sequence abundance of methanotrophs and other methylotrophs (Fig. 3B). All methyotrophic genera showed higher sequence abundance in sediments than in water, except the methanotrophic Gammaproteobacterium *Methylovulum*. Populations affiliated with that genus displayed slightly higher average sequence abundance among suspended cells, but the difference was not significant. Two signature anaerobic genera, *Pelosinus* and *Desulfosporosinus*, were more abundant among suspended cells than in attached communities (15×, 5×, respectively). This indicated the biofilms attached to sand particles did not feature steep oxygen gradients, because such gradients would have led to higher abundances of these anaerobes in attached communities. The two Gracilibacteria ASVs #24 and #74 displayed 1.5‐ and 6‐times higher abundance in water samples, respectively.

### *Role of methane, oxygen and nitrate in niche differentiation*

**Figure 4:**
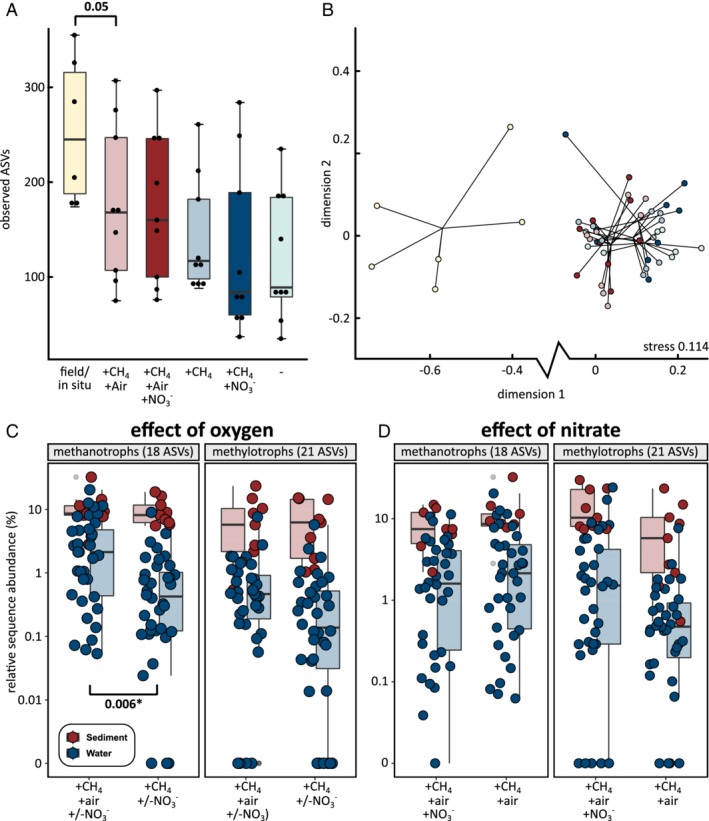
Differences between microbial communities incubated with or without methane, oxygen and nitrate. A, Sediment community richness in the incubations was lower than in the field, but differences between conditions were not significant (Wilcoxon rank sum test). B, Nonmetric multidimensional scaling (NMDS) plot showing mesocosm communities were different from field communities but similar to each other across all experiments, independent of conditions (colors are the same as in panel a). C, Relative sequence abundance of methanotrophs was higher in experiments with oxygen (Kruskal‐Wallis rank sum test). Sequence abundances of other methylotrophs were not significantly different. D, No significant differences in sequence abundances of methanotrophs and other methylotrophs were observed between experiments with and without nitrate. [Color figure can be viewed at http://wileyonlinelibrary.com]

Differences in substrate ranges and affinity among taxa and strains are often invoked to explain differences in environmental abundances and are a key aspect of canonical niche definitions of bacteria (Dunfield *et al*., 1999; Dunfield and Conrad, 2000; Ho *et al*., 2012; Hoefman *et al*., 2014; Knief, 2015). In our study, we expected that methane, oxygen and nitrate availability would be key drivers of community composition. Surprisingly, sets of replicated mesocosms did not display significant differences in overall richness (Fig. 4A) nor composition (Fig. 4B). Community richness across all mesocosms was significantly lower than in the field. Mesocosms supplied with dissolved air displayed slightly higher diversity than those without. Mesocosms supplied with nitrate displayed slightly lower diversity than those without, but both differences were not significant. Nonmetric multidimensional scaling showed communities overlapping with each other, indicating that similar communities were enriched in all mesocosms, independent of environmental conditions.

Oxygen availability increased the relative sequence abundance of methanotrophs, but not of other methylotrophs (Fig. 4C). Availability of nitrate did not significantly change the overall sequence abundances of methanotrophs and other methylotrophs (Fig. 4D). *Methylobacter*, *Methyloversatilis* and *Hyphomicrobium* sequence abundances where much higher in mesocosms with both dissolved air and nitrate, whereas *Methylocystis* abundances were highest in mesocosms with dissolved air only. Among other methylotrophs, *Methylotenera* sequence abundances were relatively stable across conditions. Although individual ASVs associated with a single methylophilic genus showed slightly different trends, no consistent patterns were observed that pointed to niche differentiation among variants within genera.

The sequence abundance of the anaerobic signature genera, *Pelosinus* and *Desulfosporosinus* were negatively affected by both air and nitrate. These bacteria were most abundant in the data sets of mesocosms without methane, nitrate and air.

The two Gracilibacteria ASV abundances displayed different responses to environmental conditions. #24 was present at much higher sequence abundance in mesocosms with dissolved air. #74 had almost even sequence abundances across all five sets of mesocosms.

### *Community turnover and succession*

**Figure 5:**
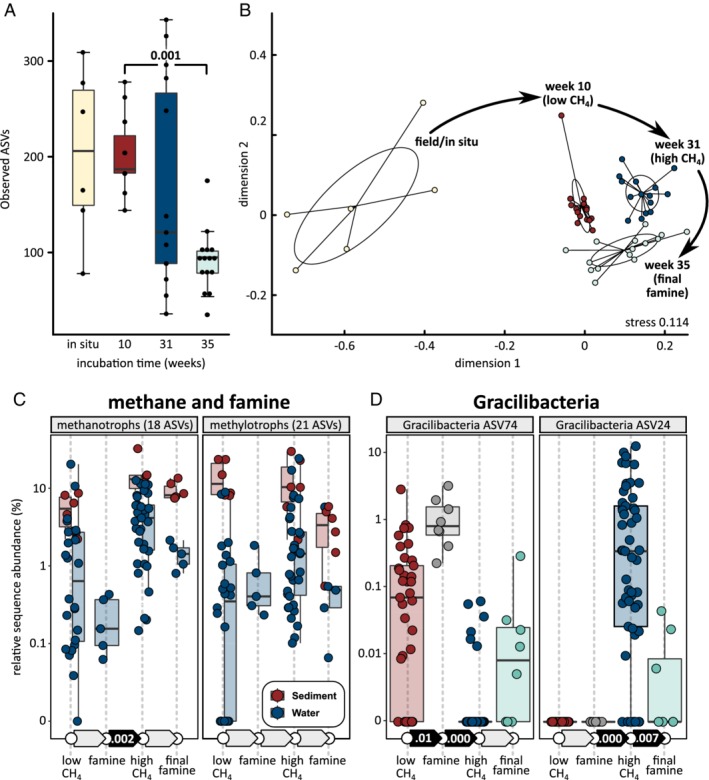
Changes of microbial community structure with incubation time. A, Sediment community richness decreased during the incubations. B, Nonmetric multidimensional scaling plot showing sediment communities displayed similar turnover across all experiments, independent of oxygen, methane or nitrate availability (Anosim R 0.68, significance 0.001). Note that panels A and B include the control, which received no methane, oxygen or nitrate. C, Relative sequence abundance of methanotrophs increased during high methane supply. The absence of methane (famine) periods did not lead to significant decline of methanotrophs. Changes in the abundance of other methylotrophs were not significant. D, Succession of two different Gracilibacteria (Candidate Phyla Radiation) populations, observed in the water (suspended cell) community. Significant differences (Kruskal‐Wallis rank sum test) between sequential time periods in C and D shown by solid black arrows with P values. Each dot represents one sample. [Color figure can be viewed at http://wileyonlinelibrary.com]

After transplantation of a natural community into a laboratory environment, adaptation and/or acclimat(izat)ion will occur, and these processes may affect community functions such as methane oxidation (Poursat *et al*., 2019). This can also lead to turnover and succession of individual populations. Over time, the mesocosms experienced a loss of richness (Fig. 5A). During the 35 week experiments, about 50 % of the observed ASVs were lost. Time displayed much stronger control over community structure than differences in environmental conditions between sets of mesocosms (Fig. 5B), as shown by clearly separated clusters for each phase of the experiments in the nonmetric multidimensional scaling plot (Anosim R 0.68, significance 0.001). Because this turnover also occurred in mesocosms without methane, oxygen or nitrate, it appeared to be unrelated to differences in methane, oxygen and nitrate availability experienced by the other mesocosms.

Weeks of famine did not lead to significant changes in relative sequence abundances for methanotrophs and other methylotrophs (Fig. 5C). However, methanotroph sequence abundance increased significantly during the “high CH_4_” phase. Among methanotrophs, *Methylobacter* displayed the highest increase in relative sequence abundance. *Methylovulum* displayed a slight decrease in relative sequence abundance during this phase. Because the latter population included a relatively large amount of suspended cells (Fig. 3C), it might have been partially “washed out” of the mesocosms at the higher medium flow rate during the “high CH_4_” phase. Among other methylotrophs, *Hyphomicrobium* displayed the highest increase in relative sequence abundance during the “high CH_4_” phase, while *Methylotenera* populations collapsed. Both signature anaerobic genera displayed much higher sequence abundances during the “high CH_4_” phase, consistent with the onset of oxygen limitation (Fig. 2E). *Methyloversatilis* was unique among all methylotrophs in that it maintained a high abundance in the amplicon data sets of mesocosms without methane.

Both Gracilibacteria ASVs displayed strong temporal dynamics (Fig. 5D). ASV #74 was abundant during the “low CH_4_” phase, bloomed during the first famine phase, and then its population collapsed for the remainder of the experiment. ASV #74 was succeeded by #24. ASV #24 was undetectable at first, but bloomed during the “high CH_4_” phase. Its population collapsed during the second famine phase.

## Discussion

The U.S. Government recommends 10 mg L^‐1^ (0.6 mM) dissolved methane as the safety threshold value, above which action must be taken due to the risks of explosion involved with out‐gassing of methane and its accumulation (Eltschlager *et al*., 2001; Humez *et al*., 2016). During the first ten weeks of our incubations, the mesocosms received medium containing 0.2‑0.4 mM dissolved methane. Under laboratory conditions, this mimicked a minor shallow aquifer methane contamination event. During this time, the measured dissolved oxygen concentration (0.01‐0.13 mM) was generally sufficient to realize complete methane oxidation.

Between weeks 19 and 33, the mesocosms were supplied with up to 1 mM or 16 mg L^‐1^dissolved methane, mimicking a contamination above the safety threshold. This led to incomplete, aerobic oxidation of methane. In our previous field study (Cahill *et al*., 2017), anaerobic methane oxidation did not appear to play a role in methane bioremediation, and the same was true in the present study. Because subsurface methane release displaces oxygen, and anaerobic methane oxidation can apparently not be taken for granted, methane bioremediation can become a slow process, taking hundreds of days, as was previously shown (Cahill *et al*., 2017). Future studies may show to what extent it is possible to establish anaerobic methane oxidation by bioaugmentation approaches (Takuechi *et al*., 2004; Nikolopoulou *et al*., 2013; Dai *et al*., 2015).

Even in the absence of anaerobic methanotrophs, it was still surprising that nitrate (supplied at 0.3 mM, 20 mg L^‐1^) did not significantly improve methane bioremediation. Nitrate occurs naturally in groundwater typically at concentrations <10 mg L^‐1^, with higher concentrations attributed to anthropogenic contamination from synthetic fertilizers or manure in agricultural runoff (Rouse *et al*., 1999; Wassenaar *et al*., 2006; Canadian Council of Ministers of the Environment, 2012; Sebilo *et al*., 2013) or wastewater sources. Its maximum allowable concentration (MAC) for drinking water according to the Canadian Water Quality Guidelines (CWQG) for the protection of aquatic life is 45 mg NO_3_^‐^ L^‐1^ (Canadian Council of Ministers of the Environment, 2012). Aerobic methanotrophs have been observed to shift to a partially anaerobic metabolism, with nitrate replacing oxygen as terminal electron acceptor for the later steps of the methanotrophic pathway (Hoefman *et al*., 2014; Kits *et al*., 2015; Heylen *et al*., 2016). Aerobic methanotrophs are also known to hand off metabolites to other methylotrophs (Nercessian *et al*., 2005; Chistoserdova *et al*., 2009; Takeuchi, 2019). These then assimilate and/or further oxidize these metabolites with nitrate as electron acceptor (Mustakhimov *et al*., 2013). Methanotrophs and their methylotrophic associates could also compete for oxygen under aerobic or oxygen‐limiting conditions.

Across all experiments and samples, the relative sequence abundance of methanotrophs and other methylotrophs were quite similar. This indicated that either a large part of the methane was transferred from the methanotrophs to the other methylotrophs in the form of metabolites such as methanol and acetate, and/or that the other methylotrophs were more versatile, feeding on substrates from other sources. The persistence of *Methyloversatilis* in experiments without methane was consistent with the latter possibility (Kalyuzhnaya *et al*., 2006). In any case, the overall methylotroph abundance was not significantly stimulated by nitrate and nitrate did not enhance methane bioremediation. Rates of microbial nitrate reduction were observed to be somewhat erratic and non‐reproducible in our mesocosms, despite all other conditions being well constrained and controlled. *Methylobacter*, *Methyloversatilis* and *Hyphomicrobium* might have consumed nitrate, because they appeared to benefit the most from nitrate addition.

The replicated, controlled experimental design provided some support for niche differentiation among methanotrophs and other methylotrophs. *Methylobacter* was found to benefit from both oxygen limitation and nitrate (SIMPER *p* < 0.05). This bacterium was the most abundant in the field methane release experiment, which was characterized by oxygen‐limiting conditions. Among other methylotrophs, *Methyloversatilis* and *Hyphomicrobium* appeared to benefit most from nitrate, even though *Methylotenera* could in theory also compete for nitrate (Mustakhimov *et al*., 2013). *Methyloversatilis* was most successful in the absence of methane. All these observations are consistent with the current understanding of methylotroph physiology (Kalyuzhnaya *et al*., 2006; Ho *et al*., 2012; Knief, 2015). Thus, amplicon sequencing could enable inferences about environmental conditions based on known ecophysiological niches of detected taxa.

Ecological selection by methane, oxygen and nitrate concentrations occurred against a background of community turnover that exerted much stronger pressure on overall community composition and was independent of environmental conditions. This might explain the variability in methylotroph abundances between replicates and shows that ecological interactions, including antagonistic interactions between microbial populations and viral predation, might be more important factors for ecological success than consistent differences in ecophysiological niches.

The ecological success of a population affiliated with Gracilibacteria is the clearest example of the importance of ecological interactions in our study. These bacteria also bloomed during our previous field methane release experiment, after methane injection was stopped (Cahill *et al*., 2017). Thus, in both field and mesocosm studies, a period of famine appeared to stimulate growth of these bacteria. *Gracilibacteria* is part of the Candidate Phyla Radiation (CPR) (Brown *et al*., 2015; Hug *et al*., 2016), comprised uncultured bacteria, ubiquitous in groundwater, and having “incomplete” central metabolism (Wrighton *et al*., 2012; Hanke *et al*., 2014; Dudek *et al*., 2017). Although their metabolic repertoire is very sparse, the genomes of some *Gracilibacteria* have been shown to encode several proteins of the serine pathway for formaldehyde assimilation and amino acid biosynthesis (Hanke *et al*., 2014) including serine hydroxymethyltransferase, formate‐tetrahydrofolate ligase, and bifunctional protein FolD. Among CPR Bacteria, *Gracilibacteria* have been shown to use a different genetic code and are believed to survive through a highly syntrophic or parasitic lifestyle (Hanke *et al*., 2014; Anantharaman *et al*., 2016; Probst *et al*., 2018). Cross *et al*. (2019) showed that related SN01 bacteria may be isolated by immuno‐targeting cell surface proteins used by SN01 for attachment to prey bacteria. The blooming of these bacteria during famine periods indicates they may at least benefit from the demise of other populations.

Our study shows that a lack of detection of methanotrophs is a clear indicator for the absence of significant methane oxidation. However, because of their persistence, the presence of these organisms does not necessarily indicate that methane is currently being oxidized. Instead, their presence might also result from persistence after past methane‐release events. Our results further indicate that obtaining sediment cores would provide a much more realistic assessment of methylotroph abundances than relying solely on water samples from groundwater wells.

Stable isotopes compositions of methane, carbon dioxide and nitrate proved extremely useful to demonstrate biological conversions and are highly recommended as a complimentary tool in field studies, where mass balances will be less useful to infer biological consumption. The methane used in our study was of thermogenic origin with a δ^13^C value of ‐36 ‰. Following methane oxidation, the δ^13^C value of the residual methane was up to 10 ‰ higher, indicating an even stronger thermogenic methane isotope signature. Our results also clearly showed that occurrence of methanotrophy can mask biogenic isotope signatures of methane, as it results in the ^13^C enrichment of any remaining methane. This could lead to the incorrect inference of thermogenic origins for biogenic methane in groundwater surveys (Whiticar, 1999).

In conclusion, our study showed that methane bioremediation can be strictly dependent on oxygen availability, consistent with previous field work (Cahill *et al*., 2017). Nitrate, at a low, environmentally meaningful concentration, and oxygen limitation favoured growth of bacteria related to *Methylobacter*, the most abundant methanotroph in the field experiment. However, consumption of nitrate was episodic and overall, did not significantly stimulate methane oxidation. Large differences were observed between abundances of suspended and attached cells, with methanotrophs generally more abundant in attached biomass. Methane oxidation resulted in enrichment of ^13^C in residual methane, making this a strong signature for successful bioremediation, applicable to field studies. Methylotrophic populations were shown to persist in the absence of methane for many weeks. This means that detection of such bacteria in groundwater could point to active as well as a past methane contamination event. This study will guide interpretation of future field studies on microbial methane bioremediation in groundwater and provides increased understanding on the ecophysiology of methylotrophic bacteria with regard to varying oxygen, methane and nitrate concentrations.

### *Experimental procedures*

*Mesocosms*. Five sets of triplicated mesocosms were used for experiments. Each mesocosm consisted of a sand‐packed, fused quartz column (inner diameter 15 mm, outer diameter 18 mm, height 400 mm, volume 71 ml). It was closed with a butyl rubber stopper at the top and bottom, which was fitted with a needle for supply and removal of medium (Fig. 1). The sand (premium play sand, The QUIKRETE Companies, Atlanta, GA, USA) was screened with a mesh No.40 to retain sand with particle size between 0.42 mm and 1 mm. Afterwards, the sand was washed five times with ample sterile deionized water, sterilized by autoclaving at 121°C for 20 min and dried in an oven at 105°C. The mesocosm experiments were performed over 35 weeks (Fig. 1).

*Mesocosm inoculation*. The inoculum was obtained between November 2015 and June 2016 from a well‐characterized shallow freshwater aquifer research facility located at the Canada Forces Base (CFB), Borden, Ontario, Canada (Cherry *et al*., 1983; Sudicky and Illman, 2011). The groundwater samples were collected during a controlled shallow groundwater methane release experiment (Cahill *et al*., 2017) at depths between 2 and 8 m below a 1 m vadose zone from two monitoring wells M6 and M7. These two wells were located 1 m apart in the downstream groundwater flow direction from the methane injection point (Cahill *et al*., 2017). Sterile 1 L Nalgene HDPE bottles were completely filled with groundwater and shipped in iced coolers to Calgary. Upon arrival (5‐7 days after sampling), DNA extraction and Illumina 16S rRNA gene amplicon sequencing was performed on 250 ml aliquots of each sample. The remainder was stored for 9‐14 months at 4°C until inoculation. Seven samples with a combined volume of 5 L were pooled into a sterile bottle and recirculated (0.5 Lday^‐1^) over five triplicated mesocosms for 72 h. The direction of flow through the mesocosms was from bottom to top, to displace air pockets out of the mesocosms during inoculation.

*Incubation media*. All mesocosms were continuously supplied with a mineral salts medium (Whittenbury *et al*., 1970), containing (g L^‐1^): MgSO_4_∙7H_2_O 1.0, CaCl_2_.6H_2_O 0.2, KH_2_PO_4_ 0.27, Na_2_HPO_4_.2H_2_O 0.72 and NH_4_Cl 0.002. It also contained 0.5 mL L^‐1^ of a solution containing (g L^‐1^) ferric ammonium citrate 1.0, sodium ethylene‐diamine‐tetra‐acetate (EDTA) 2.0, 38% hydrochloric acid 3 mL L^‐1^. Finally, it contained 1 ml of (g L^‐1^) sodium EDTA 0.5, FeSO_4_.7H_2_O 0.2, ZnSO_4_.7H_2_O 0.01, MnCl_2_.4H_2_O 0.003, H_3_BO_3_ 0.03, CoCl_2_.6H_2_O 0.02, CaCl_2_.2H_2_O 0.001, NiCl_2_.6H_2_O 0.002 and Na_2_MoO_4_.2H_2_O 0.003. Nitrate was added as KNO_3_ as specified in the results section. This medium was prepared in magnetically stirred 10 L Schott bottles with Teflon lids. Media vessels for the mesocosm experiments were sparged continuously, during the entire experiment, using mass flow controllers (Alicat Scientific, Tucson, AZ, USA), with a mixture of air, methane, and helium. Sparging started 24 h before medium was supplied to the mesocosms. The medium vessels were stirred at 100 rpm. Gas was vented from the medium bottles via a water lock to prevent overpressure and backflow of air into the bottles (Fig. 1). Triplicated mesocosms were supplied with sterile medium from the same feed bottle at a rate of 100 mL day^‐1^ (~1.4 mesocosm volume changes day^‐1^, ~1.8 m day^‐1^) during the first seventeen weeks. Between weeks 17 and 25, the rate was gradually increased to 200 mL day^‐1^, maintained until the end of the experiment. Media was pumped into the top of the mesocosm experiments using peristaltic pumps (Ismatec, Wertheim, Germany). Effluent (spent) medium from the mesocosms was collected in a 10 L Schott bottle. All mesocosms were maintained at room temperature (23°C). All columns were covered with black polyethylene sheeting to prevent growth of phototrophs.

*Measurement of dissolved oxygen concentrations and pH*. Dissolved oxygen concentrations were measured using oxygen sensor spots (OXSP5; Pyroscience, Aachen, Germany) near the top and bottom of mesocosm columns. The sensor spots were connected to a FireSting O_2_ fiberoptic oxygen meter (Pyroscience) previously calibrated to 100% and 0% oxygen according to manufacturer's instructions. Media supplied to the mesocosms was also monitored at regular intervals for dissolved oxygen with an inline flow‐through cell fitted with such a sensor spot. The sensor measures oxygen using red light excitable materials that generate oxygen‐dependent luminescence in the near infrared. The pH of inflowing and outflowing medium was measured offline with a benchtop pH meter (Mettler Toledo, Columbus, OH, USA).

*Sample collection from mesocosms*. Effluent (spent) medium from all mesocosms was collected in 50 mL falcon tubes covered with autoclaved aluminum foil, overnight on ice. A 35 mL aliquot of the collected effluent was filtered through a 0.1 μm VCTP membrane filter (MilliporeSigma, Billerica, MA, USA) attached to a sterile 15 mL glass microanalysis filter holder (MilliporeSigma). The remainder was used to determine microbial cell numbers (see below). For DNA extraction from mesocosm sediments in weeks 10, 31 and 35, the mesocosms were opened at the top and approximately 1 g of sediment (~ 20 mm) was removed from each mesocosm with a sterile spatula and transferred into a 15 mL falcon tube. All filters and sediments were stored at ‐20°C until DNA extraction. For measurements of dissolved gases, autoclaved, helium‐flushed serum bottles (30 mL) were first filled completely with the same media used for a given mesocosm. This bottle was then placed between the medium vessel and the mesocosm. It was fitted with a long 21 Gauge 0.8 × 50 mm needle (BD, Franklin Lakes, NJ, USA) and a short 25 Gauge 0.5 × 25 mm needle (BD) such that inflowing media first passed through this serum bottle before it entered the mesocosm. A similar serum bottle (filled with deionized water instead of medium and chilled at 0°C) was placed between the mesocosm and the effluent vessel. The serum bottles were left to equilibrate for at least 15 hours to ensure that approximately five serum bottle volumes had flowed through. Then, serum bottles were disconnected, and stored at 4°C until analysis, within 24 h after sample collection.

*Concentration and isotope ratio measurements*. Dissolved gas concentrations (CH_4_, C_2_H_6_, CO_2_, N_2_ and O_2_) were analyzed after separation from water using the static headspace equilibrium technique (Kampbell and Vandegrift, 1998) and measured on a Bruker 450 Natural Gas chromatograph with measurement uncertainties of ± 5%. Stable carbon isotope (δ^13^C, relative to VPDB, Vienna PeeDee Belemnite) ratios of CH_4_ and CO_2_ were analyzed on a MAT 253 isotope ratio mass spectrometer (IRMS) coupled to Trace GC Ultra and GC Isolink (Thermo Fischer Scientific, Waltham MA, USA), with an error of <0.5‰ for CH_4_ and 0.3‰ for CO_2_. The nitrate concentration was measured as total oxidized nitrogen using a Gallery Plus automated photometric analyzer (Thermo Fischer Scientific). The isotopic composition of nitrate was determined on N_2_O generated by the denitrifier technique (Casciotti *et al*., 2002, 2007), using a Delta V Plus IRMS coupled to a Finnigan MAT PreCon (Thermo Fischer Scientific), with an accuracy of 0.3‰ and 0.7‰ for δ^15^N‐NO_3_ and δ^18^O‐NO_3_, respectively.

*Microbial cell numbers*. Unfiltered effluent water samples (0.5 mL) were mixed with 1.5 mL of sterile 1% phosphate buffered saline (PBS) and 108 μl of filter‐sterilized 37% formaldehyde. The fixed samples were stored overnight at 4°C and subsequently filtered through a sterile 0.1 μm MilliporeSigma VCTP membrane filter. The filters were washed twice with 1% PBS and dried with 1% PBS/ethanol (1:1) solution. The filters were stored at ‐20°C until analysis. The cells were stained with DAPI (4', 6‐diamidino‐2‐phenylindole) as described previously (Porter and Feig, 1980). The filters were viewed under a Zeiss Axio Imager A.2 microscope (Carl Zeiss Microscopy GmbH, Jena, Germany) equipped with a X‐Cite 120 LED lamp (Lumen Dynamics, Mississauga ON, Canada) and Zeiss Axiocam 506 mono camera. Cell counts were averaged for 20 fields of view (Kepner *et al*., 1994).

*DNA extraction*. DNA from the 0.1 μm filters and sediment samples was extracted with the FastDNA Spin Kit for Soil (MP BioMedicals, Santa Ana, CA, USA) according to manufacturer's instructions. Extracted DNA samples were quantified with a Qubit 2.0 Fluorometer (Thermo Fischer Scientific) and stored at ‐20°C until DNA amplification.

*Illumina 16S rRNA gene sequencing*. The V3‐V4 region of 16S rDNA was amplified in a single‐step PCR using a KAPA HiFi HotStart reaction kit and primers Pro341F/Pro805R targeting prokaryotes (Takahashi *et al*., 2014) and including Illumina adapters (Illumina Inc. San Diego, CA, USA). The primers, Pro341F (5´‐CCT ACG GGN BGC ASC AG‐3´) and Pro805R (5´‐GAC TAC NVG GGT ATC TAA TCC‐3´) complemented standard Illumina forward and reverse primers. PCR mixtures contained 0.1 μM of the forward primer, 0.1 μM of the reverse primer, 12.5 μl of 2× KAPA HiFi HotStart Ready Mix (Kapa Biosystems, Wilmington, MA, USA) and 1 μl of template DNA (~1 ng μL‐1), made up to 25 μl with nuclease‐free water. PCR reaction conditions were as follows: initial denaturation at 95°C for 3 min, followed by 32 cycles of 95°C for 30 s, 55°C for 45 s, and 72°C for 60 s, followed by a final step of 72°C for 5 min. The amplicon products of triplicate PCR reactions were pooled and purified using 0.8× volume of AMPure XP magnetic beads (Beckman Coulter, Indianapolis, IN, USA) as per manufacturer's instructions. DNA amplicon libraries were prepared from purified PCR‐products using an Illumina Nextera XT DNA Library Prep Kit (i5 adapters.) with Nextera XT index kit v2 (i7 adapters) as per the manufacturer's instructions. Reaction conditions of the second (index) PCR were as follows: 95°C for 3 min, followed by 10 cycles of 9°C for 30 s, 5°C for 45 s, and 72°C for 60 s, and a final step of 72°C for 5 min. PCR products were purified with AMPure XP beads and quantified with a Qubit 2.0 Fluorometer. Amplicon libraries were normalized to 2 nM, pooled in equal volumes, denatured in 0.2 N NaOH and diluted with hybridization buffer according to the Nextera XT protocol. Paired‐end sequencing (300 × 300 bp) of libraries at 15 pM final concentration was performed on an Illumina Miseq instrument using manufacturer's reagents and according to the manufacturer's instructions.

*Sequence Analyses*. Sequenced libraries were analyzed using *dada2* following the DADA2 Pipeline Tutorial v1.12 (Callahan *et al*., 2016). Briefly, forward reads were quality‐trimmed to 275 bp and reverse reads to 215 bp. Primer sequences (17 bp forward, 21 bp reverse) were removed from the sequence reads. Reads with more than two expected errors were discarded (“maxEE=c(2,2)”). Paired reads were merged with the default dada2::mergePairs parameters. Chimeric sequences were removed using dada2::removeBimeraDenovo and species level taxonomy was assigned using dada2::assignTaxonomy and dada2::addSpecies with silva_nr_v132_train_set and silva_species_assignment_v132, which are based on the Silva small subunit reference database SSURef v132 (release date: Dec, 13 2017; Quast *et al*., 2013). The original *dada2* output ASV‐by‐sample table was used to determine ASV richness and composition. Wilcoxon signed rank‐tests were performed with *ggsignif*, an extension to *ggplot2*. Shannon entropy was calculated from the ASV‐by‐sample table using subsampling, to account for unequal sampling. Bray‐Curtis dissimilarities (Bray and Curtis, 1957) between all samples were calculated and used for two‐dimensional nonmetric multidimensional scaling (NMDS) ordinations with 20 random starts (Kruskal, 1964). All analyses were carried out with the R statistical environment and the packages *vegan* (Dixon, 2003), *ggplot2 (*Wickham, 2009 *)*, as well as with custom R scripts.

## Supporting information


**Supplementary Data S1** (https://fig.com/articles/Figure_S1/8175473). Concentrations of methane, oxygen, nitrate, carbon dioxide in inflowing and outflowing media of all mesocosms, pH, and isotopic signatures of methane, carbon dioxide and nitrate, as well as relative sequence abundances for all amplicon sequence variants.

## Data Availability

The authors declare that the data supporting the findings of this study are available within the article and its Supplementary Information. An online version of the supplementary information is also available: (https://figshare.com/articles/Figure_S1/8175473). DNA sequence data are available in the NCBI database under accession number PRJNA513134 (https://www.ncbi.nlm.nih.gov/bioproject/PRJNA513134).
