# Supplementary material for: Methane oxidation and methylotroph population dynamics in groundwater mesocosms
Source: Environ Microbiol. 2020 Feb 7;22(4):1222–37. doi: 10.1111/1462-2920.14929 (PMC7187433; doi:10.1111/1462-2920.14929)
Supplement: Supplementary file 1 — Supplementary Data S1 (https://fig.com/articles/Figure_S1/8175473). Concentrations of methane, oxygen, nitrate, carbon dioxide in inflowing and outflowing media of all mesocosms, pH, and isotopic signatures of methane, carbon dioxide and nitrate, as well as relative sequence abundances for all amplicon sequence variants. [file EMI-22-1222-s001.docx]

**Supplementary data 1 (**[**https://figshare.com/articles/Figure_S1/8175473**](https://figshare.com/articles/Figure_S1/8175473)**).** Concentrations of methane, oxygen, nitrate, carbon dioxide in inflowing and outflowing media of all mesocosms, pH, and isotopic signatures of methane, carbon dioxide and nitrate, as well as relative sequence abundances for all amplicon sequence variants.

**Table 1 |** Taxonomic affiliation and statistics of key amplicon sequence variants (ASVs). Supplementary data 1 lists taxonomic classifications, sequences and abundances of all ASVs across all samples.

| **Physiology** | **Genus** | **Class** | **# ASVs obser-ved** | **Field average abun-dance (%)** | **Mesocosm average abundance** **(%)** | **Mesocosm maximum abundance (%)** | **#obser-vations** | **ASVs** |
| --- | --- | --- | --- | --- | --- | --- | --- | --- |
| Methanotroph | Methylocystis/sinus* | Alpha-proteobacteria | 3 | 0.0 | 2.4 | 32.3 | 196 | 15, 23, 82 |
| Methanotroph | Methylobacter | Gamma-proteobaceria | 5 | 14.5 | 0.1 | 6.1 | 56 | 55, 124, 272, 444, 1051 |
| Methanotroph | Methylovulum | Gamma-proteobaceria | 6 | 0.4 | 0.4 | 19.8 | 153 | 34, 40, 167, 402, 592, 1057 |
| Methanotroph | Methylomonas | Gamma-proteobaceria | 2 | 0.0 | 0.1 | 24.0 | 19 | 89, 187 |
| Methanotroph | Crenothrix | Gamma-proteobaceria | 2 | 0.0 | 0.0 | 0.8 | 11 | 437, 823 |
| Methylotroph | Hyphomicro-bium | Alpha-proteobacteria | 8 | 0.0 | 0.2 | 4.6 | 113 | 88, 228, 424, 853, 1115, 1227, 1831, 3223 |
| Methylotroph | Methylo-bacterium | Alpha-proteobacteria | 3 | 0.0 | 0.0 | 1.0 | 24 | 533, 710, 875 |
| Methylotroph | Methylo-versatilis | Beta-proteobacteria | 1 | 0.0 | 2.1 | 29.4 | 194 | 14 |
| Methylotroph | Methylotenera | Beta-proteobacteria | 6 | 2.1 | 0.2 | 3.6 | 122 | 106, 190, 327, 539, 646, 945 |
| Methylotroph | Methylophilus | Beta-proteobacteria | 3 | 3.0 | 0.1 | 13.5 | 14 | 111, 153, 1552 |
| Unknown | Gracilibacteria | Candidate Phyla Radiation | 2 | 0.0 | 0.6 | 12.1 | 150 | 24, 74 |
| Fermentation | Pelosinus | Negativicutes | 11 | 0.0 | 2.3 | 86.4 | 94 | 10, 19, 707, 1452, 1592, 2442, 2550, … |
| Sulfate reduction | Desulfo-sporosinus | Clostridia | 40 | 2.4 | 0.3 | 19.8 | 162 | 46, 351, 374, 381, 382, 457, 525, 724, … |

** These two genera could not be discriminated based on the 400 nucleotide amplicon.*
